# Red cell distribution width is not a predictor of mortality in acute kidney injury

**DOI:** 10.1186/cc12354

**Published:** 2013-03-19

**Authors:** P Purvis, J Kinsella, R Docking

**Affiliations:** 1 University of Glasgow, UK; 2 Glasgow Royal Infirmary, Glasgow, UK

## Introduction

Given the significant morbidity and mortality associated with acute kidney injury (AKI), there is a need to find factors to help aid decision-making regarding levels of therapeutic support. As a prognostic biomarker, the red cell distribution width (RDW) has attracted interest in the setting of critical care when added to existing scoring systems [1]. By examining RDW in a previously studied AKI cohort, we aimed to evaluate the utility of this routine blood test.

## Methods

A cohort of 209 mixed critical care patients who received renal replacement therapy for AKI had their demographic and biochemical data retrieved from electronic databases. Outcomes were gathered for ICU and hospital mortality. Incomplete datasets were discarded, leading to 153 complete sets. RDW data were taken from the first sample after admission to the ICU, as were all other biochemical values apart from pre-RRT creatinine and potassium. Overall cohort characteristics were gathered, and two groups were created: those with a RDW value within normal range (≤14.5%) and those with a greater than normal value (>14.5%). We then further subgrouped RDW to assess the correlation between rising levels and ICU mortality.

## Results

A total 77.1% of our cohort had a RDW greater than the normal laboratory range at time of ICU admission. Key baseline characteristics (age, APACHE II score, length of stay, ICU mortality) did not differ significantly between patients with normal and abnormal RDW. When subgroup analysis was performed, no statistically significant correlation between rising RDW and ICU mortality was found (Spearman correlation = 0.426, *P *= 0.233).

## Conclusion

In this cohort of critically ill patients with AKI, RDW was not found to be a predictor of mortality. Our results contradict those of recent studies [1,2]. However, both groups of RDW patients in our study suffered a higher ICU mortality than in other studies. To further explain these findings, we intend to perform multivariate logistic regression analysis and assess the effect of social deprivation on RDW.
